# Brain connectivity changes to fast versus slow dopamine increases

**DOI:** 10.1038/s41386-024-01803-8

**Published:** 2024-02-07

**Authors:** Peter Manza, Dardo Tomasi, Leah Vines, Diana Sotelo, Michele-Vera Yonga, Gene-Jack Wang, Nora D. Volkow

**Affiliations:** National Institute on Alcohol Abuse and Alcoholism, National Institutes of Health, Bethesda, MD USA

**Keywords:** Reward, Addiction

## Abstract

The rewarding effects of stimulant drugs such as methylphenidate (MP) depend crucially on how fast they raise dopamine in the brain. Yet how the rate of drug-induced dopamine increases impacts brain network communication remains unresolved. We manipulated route of MP administration to generate fast versus slow dopamine increases. We hypothesized that fast versus slow dopamine increases would result in a differential pattern of global brain connectivity (GBC) in association with regional levels of dopamine D1 receptors, which are critical for drug reward. Twenty healthy adults received MP intravenously (0.5 mg/kg; fast dopamine increases) and orally (60 mg; slow dopamine increases) during simultaneous [^11^C]raclopride PET-fMRI scans (double-blind, placebo-controlled). We tested how GBC was temporally associated with slow and fast dopamine increases on a minute-to-minute basis. Connectivity patterns were strikingly different for slow versus fast dopamine increases, and whole-brain spatial patterns were negatively correlated with one another (rho = −0.54, *p*_spin_ < 0.001). GBC showed “fast>slow” associations in dorsal prefrontal cortex, insula, posterior thalamus and brainstem, caudate and precuneus; and “slow>fast” associations in ventral striatum, orbitofrontal cortex, and frontopolar cortex (*p*_FDR_ < 0.05). “Fast>slow” GBC patterns showed significant spatial correspondence with D1 receptor availability (estimated via normative maps of [^11^C]SCH23390 binding; rho = 0.22, *p*_spin_ < 0.05). Further, hippocampal GBC to fast dopamine increases was significantly negatively correlated with self-reported ‘high’ ratings to intravenous MP across individuals (*r*_*(*19)_ = −0.68, *p*_bonferroni_ = 0.015). Different routes of MP administration produce divergent patterns of brain connectivity. Fast dopamine increases are uniquely associated with connectivity patterns that have relevance for the subjective experience of drug reward.

## Introduction

The faster a drug enters the brain, the more rewarding it is. For instance, when stimulant drugs such as methylphenidate (MP) are delivered via routes that result in fast brain delivery (e.g., intravenous injection), they produce a greater ‘high’ (i.e., euphoria) than when delivered via routes that result in slower brain delivery (e.g., orally) [1]. These effects are in large part due to the speed at which these routes of administration increase striatal dopamine [2]. In rats, speedier delivery of cocaine produces a faster rise in dopamine and, accordingly, greater cocaine self-administration [3–5]. Likewise, in humans, intravenous compared to oral drug administration produces faster increases in striatal dopamine [6], and chronic drug misuse via faster routes of administration are associated with faster transition to substance use disorder (SUD) and more severe symptomatology [7, 8]. These studies support the theory that fast dopamine increases elicit neuroplastic changes in large-scale brain circuits underlying reward, promoting further drug-seeking behavior [1]. Therefore, finding circuits sensitive to rate of dopamine increases is a key step in understanding the pathophysiology of SUD.

Early human studies using positron emission tomography (PET) confirmed that faster routes of MP administration produced a greater and more consistent ‘high’ than routes of administration that resulted in slower delivery [9–12]. However, these studies could not identify the downstream circuit effects of fast dopamine increases, and how these contributed to the experience of drug reward. In a recent study we utilized simultaneous PET-fMRI [13] while healthy adults were administered MP orally (triggering slow dopamine increases) and intravenously (triggering fast dopamine increases) to begin exploring this phenomenon [14]. We found that both slow and fast dopamine increases decreased fMRI activity in ventromedial prefrontal cortex. However, only fast dopamine increases activated the ‘salience network’, including dorsal anterior cingulate cortex and insula.

This finding was an important first step, yet regional fMRI activation patterns only describe part of the complex network response to MP. Further, it remains unclear how network connectivity changes may predict individual differences in the subjective experience of drug reward, a measure with predictive value for future substance misuse [15]. Global brain connectivity (GBC; also known as ‘weighted degree’ [16, 17]) is a marker of whole-brain network communication with strong test-rest reliability [18] that is well-suited to address these issues: GBC exhibits (a) neurobiological relevance, showing strong association with target receptor stimulation in response to drugs and (b) behavioral relevance, showing strong association with individual differences in the subjective experience to drugs [19, 20]. There are also methodological advantages to GBC because it requires no a priori seed region selection, and it can be examined at voxel level or parcellated into large-scale networks that support healthy brain function. A recent large-scale study showed that GBC changes in response to 10 different psychoactive drugs depended on the spatial distribution of each drug’s target receptor and/or transporter density [21]. Thus, GBC is an excellent candidate to further explore more complex patterns of network communication in response to fast and slow dopamine increases.

Here, we re-analyzed data from our prior PET-fMRI study and tested how the rate of dopamine increases impacts GBC. We hypothesized that slow and fast dopamine increases would elicit different patterns of GBC because they would stimulate different dopamine receptor subtypes that have unique spatial distributions. Specifically, whereas slow dopamine would primarily stimulate high-affinity D_2_ receptors, fast dopamine should additionally stimulate the lower-affinity D_1_ receptors [22–24]. In line with this, we hypothesized that GBC patterns associated with fast compared to slow dopamine increases would show spatial correspondence with normative maps of D_1_ receptors. Finally, in exploratory analysis, we tested how GBC patterns in functional brain networks correlated with individual differences in self-reports of ‘high’ to MP.

## Methods and materials

Some of the PET data and the behavioral data (‘high’ ratings) were previously reported in a recent publication [6]. Here, we investigated the relationship between dynamic dopamine changes and brain functional connectivity assessed with fMRI. All of the primary fMRI results in this manuscript are novel and have not been published.

### Participants

Twenty healthy individuals (36.1 ± 9.6 years old; 9 females) participated in the study (see Supplementary Table 1 for participant characteristics). Participants were recruited through referrals from the NIH Volunteer Office, the Patient Recruitment and Public Liaison (PRPL) Office, ResearchMatch.org, by word of mouth, and through Institutional Review Board (IRB)-approved advertisements. All individuals provided informed consent to participate in this double-blind placebo-controlled study, which was approved by the IRB at the National Institutes of Health (Combined Neurosciences White Panel; Protocol 17-AA-0178). This study was registered at clinicaltrials.gov (trial NCT03326245 on October 31, 2017). All participants self-reported no history of nicotine/tobacco use. All participants were compensated for study participation.

### Exclusion criteria

Participants were screened to exclude major medical and neuropsychiatric disorders that can impact brain function (seizures, tics, agitation, anxiety, panic attacks, mood disorders, glaucoma, neurodegenerative disorders), past or present history of substance use disorders (lack of drug use was confirmed with a urine drug screen for benzodiazepines, cocaine, methamphetamines, opiates and tetrahydrocannabinol on all scan days), heart abnormalities (confirmed with electrocardiography), hypertension requiring medication or arrhythmia, pregnancy (confirmed with a urine pregnancy test) or breastfeeding, medications that may interact with methylphenidate (stimulants, analgesics containing narcotics, anorexics, antianginal agents, antiarrhythmics, corticosteroids, antibiotics, anticholinergics, anticoagulants, anticonvulsants, antidepressants, antidiarrheal, antifungal, antihistamines, antihypertensives, anti-inflammatory; antineoplastics, antiobesity, antipsychotics, antivirals, anxiolytics, hormones, insulin, lithium, muscle relaxants, psychotropic drugs, sedatives/hypnotics), or ferromagnetic body implants that are contraindicated for MRI.

### Experimental design

**Fig. 1:**
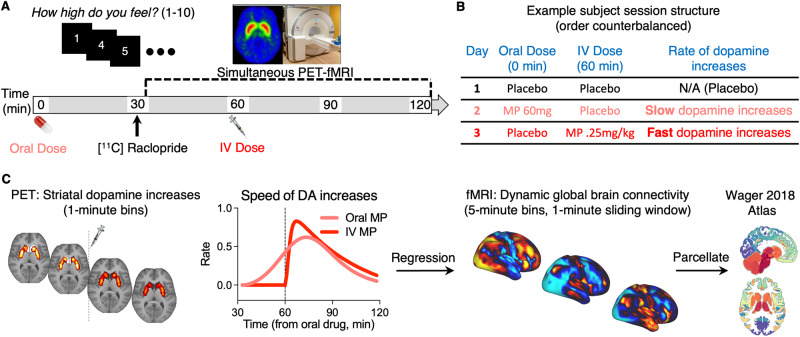
Experimental design. **A** Timeline of events. In each session, participants were given an oral dose of methylphenidate (MP) or placebo at time 0; the [^11^C] Raclopride bolus injection and simultaneous PET-fMRI scanning started at 30 min; an IV dose of MP or placebo was given at 60 min; and throughout the duration of the session participants used a button box in the scanner to self-report their experience of ‘high’ to the drug. **B** An example subject’s session structure. Participants three separate imaging sessions on different days. Sessions were identical except for drug condition: (Session 1) oral PLA and IV PLA; (Session 2) oral MP (60 mg) and IV PLA (3 cc saline); (Session 3) oral PLA and IV MP (0.25 mg/kg in 3 cc sterile water). The session order was counterbalanced across participants. **C** Analysis schema. With PET data, minute-to-minute changes in dopamine receptor binding were used to estimate the rate of dopamine increases in response to MP (decreases in D2 receptor binding following MP are a proxy for dopamine increases). With fMRI data, we calculated minute-to-minute changes in global brain connectivity across the scan session. We then performed multiple regression to identify global brain connectivity patterns that were temporally associated with slow and fast dopamine increases. Finally, we parcellated these maps to show how dopamine rate-associated patterns of global brain connectivity were distributed across large-scale brain functional networks.

The procedure for the study is illustrated in Fig. 1A. Each participant was scanned on 3 different days, 40 ± 35 days apart, under different pharmacological conditions: (1) oral-MP (60 mg) and iv-placebo (3 cc saline), (2) oral-placebo and IV-MP (0.25 mg/kg in 3 cc sterile water), and (3) oral-placebo and iv-placebo. The session order was randomized and blocked across every six participants (for an example subject’s session order, see Fig. 1B). Participants and the research staff were blind to medication (MP or PL) or route of administration (oral or IV). The key to the session order was held by independent personnel at the NIH Clinical Center Pharmacy until trial completion. Data were collected at the NIH Clinical Center in Bethesda, Maryland from January 2018 to September 2021.

### PET/MRI acquisition

The participants underwent simultaneous PET/MRI imaging in a 3 T Biograph mMR scanner (Siemens; Medical Solutions, Erlangen, Germany). All studies were initiated at noon to minimize circadian variability. Venous catheters were placed in the left dorsal hand vein for radiotracer injection, and in the right dorsal hand vein for intravenous injection of medications. Heart rate (HR), systolic and diastolic blood pressures (BPs) were continuously monitored throughout the study with an Expression MR400 patient monitor (Philips, Netherlands). Thirty minutes before tracer injection, either 60 mg of MP or placebo was administered orally (p.o). The participant was then positioned in the scanner. Earplugs were used to minimize scanner noise and padding to minimize head motion. A T1 weighted dual-echo image was collected for attenuation correction using an ultrashort-TE (UTE) sequence (192 × 192 × 192 matrix, 1.56 mm isotropic resolution, TR = 11.94 ms, TE = 0.07 and 2.46 ms) for PET attenuation correction, and T1-weighted 3D magnetization-prepared gradient-echo (MPRAGE; TR/TI/TE = 2200/1000/4.25 ms; FA = 9°, 1 mm isotropic resolution) was used to map brain structure. List mode PET emission data were acquired continuously for 90 min and initiated immediately after a manual bolus injection of [^11^C]raclopride (dose =15.7 ± 1.9 mCi; duration 5–10 s). Simultaneously, fMRI data were acquired continuously for 90 min with a single-shot echo planar imaging (EPI) sequence (TE/TR = 30/3000 ms, FOV = 192 × 192 mm, in-plane resolution = 3 × 3 mm, 1800 volumes, 36 slices/volume, slice thickness = 4 mm). Thirty minutes after [^11^C]raclopride injection, either 0.25 mg/kg MP or placebo was manually injected i.v. as a ~30-s bolus. The participants were instructed to stay as still as possible and keep their eyes open during the scan.

### High ratings

High rating prompts were displayed on a projector using a program (E-Prime Version 3.0) designed to minimize visual stimulation. A white cross was presented at central fixation on a black screen. Participants were instructed to stay awake, relax, look at the cross, and not think of anything in particular. Occasionally, the cross would turn into a number for 10 s, and participants responded to the question: “How high do you feel right now, on a scale of 1–10, with 1 being minimum and 10 being maximum?”. The first number presented at the start of each scanning session was always 1, and subsequent presentations matched the participant’s high rating from the prior time point. Participants used a button box in their right hand to record responses. A button pressed with the right middle finger moved the rating up, one digit at a time, whereas the other button pressed with the right index moved it down. High rating prompts occurred every 5 min from the onset of oral MP administration; then, at the onset of IV-MP administration, prompts occurred every minute for 20 min. This faster sampling was chosen to capture the fast changes in reward during the first 20 min after IV-MP administration [25]; then, prompts occurred every 5 min until the end of scanning.

### MRI preprocessing

The minimal preprocessing pipelines of the Human Connectome Project (HCP) [26] were used for image processing. Specifically, FreeSurfer 5.3.0 (http://surfer.nmr.mgh.harvard.edu) was used for automatic segmentation of anatomical MRI scans into cortical and subcortical gray matter ROIs [27]. Then, for the EPI images, the FSL Software Library (version 5.0; http://www.fmrib.ox.ac.uk/fsl) [28] was used for rigid body realignment, field map processing, co-registration to the anatomical T1 image, and spatial normalization to MNI space.

We further processed the EPI images for fMRI analysis, including: regression of white matter, CSF, global signals, head motion parameters, and their derivatives using custom MATLAB code; and 5 mm full-width at half-maximum spatial smoothing, using FSL. We also bandpass filtered the fMRI data in the 0.01–0.1 Hz frequency range.

### PET image reconstruction

A 3-dimensional ordered-subset expectation-maximization (OSEM) algorithm [29] with 3 iterations, 21 subsets, an all-pass filter, 344 × 344 × 127 matrix, and a model of the point spread function of the system was used for PET image reconstruction. The reconstructed PET time series consisted of 48 time windows (30 frames of 1 min, followed by 12 frames of 2.5 min, and 6 frames of 5 min) each with 2.086-mm in-plane resolution and 2.032-mm slice thickness. Attenuation coefficients (μ-maps) estimated from the UTE data using a fully convolutional neural network [30] were used to correct for scattering and attenuation of the head, the MRI table, the gantry, and the radiofrequency coil. Standardized uptake values (SUVs) for [^11^C]raclopride were calculated after normalization for body weight and injected dose, co-registered with the structural T1w map, and spatially normalized to MNI space using parameters obtained from the HCP pipelines [26]. Relative SUV time series, SUVr(*t*), were computed in MNI space by normalizing each SUV volume by its mean SUV in cerebellum, as defined in individual FreeSurfer segmentations.

### Statistical analysis

#### PET image analysis: estimation of dynamic ‘dopamine increases’ to oral and IV MP

Decades of clinical and preclinical research have demonstrated that [^11^C]Raclopride is sensitive to synaptic dopamine concentration, as it has lower affinity for dopamine D_2_-like receptors than endogenous dopamine [31–33]. Therefore, decreases in [^11^C]Raclopride binding following administration of a dopamine-boosting drug like MP are a suitable proxy for ‘dopamine increases’ [34, 35].

Several groups have further found that one can model the time course of [^11^C]Raclopride binding to measure the temporal dynamics of dopamine receptor occupancy (and dynamics of dopamine increases in response to dopamine-boosting interventions such as MP). Some of the most popular methods include ‘neurotransmitter PET’ (ntPET) [36], the ‘linear simplified reference region model’ (LSSRM) [37] and the ‘dynamic binding potential’ [23].

Recently, we developed a new approach to measure the temporal dynamics of dopamine changes optimally suited for the current experimental design [6], which because of the PET/MR setup, did not allow us to perform a continuous [11 C]raclopride infusion as required by LSSRM (for a demonstration of the similarities between this method and prior methods, and for advantages of the current method for this particular study design, see the Supplement, including Supplementary Fig. S1). Briefly, we found that dynamic ΔSUVr changes between placebo and MP conditions parallel the dynamics of striatal dopamine increases induced by MP as a function of time, which can be characterized by a gamma cumulative distribution function$.$ To estimate the average time-varying dopamine increases to MP in the putamen we adjusted the amplitude, *A*, and the shape, *s*, parameters of the gamma cumulative distribution function.

1 $$F\left(t\right)=\frac{A}{\Gamma (s)}{\int }_{0}^{t}{e}^{-x}{x}^{s-1}{dx},$$

to fit *F*(t) to the average ΔSUVr(t) data across the 20 participants with the Levenberg-Marquardt algorithm for non-linear least-squares fitting in the interactive data language (IDL, L3Harris Geospatial, Boulder, CO). The corresponding probability density function, *f*(*t*) = d*F*(*t*)/d*t* was used to estimate the average rate of dopamine increases at 1-min temporal resolution, independently for oral- and IV-MP, and were used as the regressors of interest for subsequent GBC analyses.

#### fMRI image analysis: GBC changes in response to slow and fast dopamine increases

We computed GBC similar to prior work [19, 20] using custom code in Python version 3.9.7 and the *nilearn* software package. For each subject, we extracted the fMRI time-series signal for each voxel in the brain, and then computed the correlation coefficient between one voxel with all other brain voxels, and Fisher Z-transformed the data. We repeated this process over every brain voxel, rendering whole-brain maps of the mean functional connectivity between one region and all other regions. To produce ‘dynamic’ GBC maps, we iterated this process across the 90-minute scanning session using a sliding window approach. Specifically, we calculated GBC across a sliding window of 5-minute bins with 4-minute overlap. This yielded a connectivity estimate for each minute of the scan (the actual number of timepoints of dynamic GBC was 82, due to inability to estimate connectivity for the first and last 4 min of the session).

To identify how brain connectivity was associated with the rate of dopamine increases to slow (oral MP) vs. fast (IV MP) drug delivery, we performed whole-brain voxelwise multiple regression analysis of dynamic GBC images in SPM. We used *f*(*t*), the PET-derived estimates of the rate of dynamic dopamine increases to oral and IV MP (average of all 20 participants), as the regressors of interest. Thus, for each individual and for the “slow dopamine increase” (oral MP) and “fast dopamine increase” (IV MP) sessions separately, we used the time course of slow and fast dopamine increases as regressors against the whole-brain voxelwise GBC maps. This temporal regression analysis yielded whole-brain maps showing where GBC was significantly associated with dynamic dopamine increases across time. We then subjected these maps (i.e., the contrast values from the multiple regression) to second-level analysis in SPM: one-sample *t* tests for each drug condition, and then paired *t* tests directly comparing fast versus slow dopamine-associated GBC maps (i.e., IV MP versus oral MP sessions). We also performed conjunction analysis to test for regions that showed overlapping GBC patterns to slow and fast dopamine increases. For whole-brain group level analyses, the significance threshold was set at voxelwise *p* < 0.001 uncorrected, with a cluster-forming threshold of *p* < 0.05 false discovery rate (FDR)-corrected, and a minimum cluster size of *k* > 50, in line with current reporting guidelines [38].

Because we hypothesized opposing connectivity patterns for fast versus slow dopamine rate-associated GBC maps (i.e., IV MP versus oral MP sessions), we formally tested the spatial correspondence between these two voxelwise images, using spatial correlations. A simple spatial correlation of one fMRI image with another is not feasible because signals from every voxel are not statistically independent—voxels that are nearby in space tend to be highly correlated with one another (i.e., fMRI connectivity images show ‘spatial autocorrelation’). Simple spatial correlations therefore yield artificially inflated correlation coefficients. To circumvent this, we used the *compare_images* command (with the *alexander_bloch* method) from the *neuromaps* package in Python [39]. The method applies random rotations to spherical representations of the cortical fMRI data to create a null distribution with a similar spatial autocorrelation structure to the empirical fMRI data [40]. Hence, we compared the spatial similarity of the maps showing the temporal association of GBC with fast dopamine increases (to IV MP) with the maps showing the temporal association of GBC with slow dopamine increases (to oral MP). The null distribution was created by permuting the fast dopamine rate-associated GBC maps 100 times. The spatial correlation of fast versus slow dopamine rate-associated GBC maps was deemed significant if it exceeded the top 5% of correlation coefficients in the null distribution.

We also hypothesized that fast compared to slow dopamine rate-associated GBC maps would be spatially correlated with the density of dopamine D1 receptors across the brain, since fast dopamine increases are more likely to stimulate low-affinity D1 receptors than slow dopamine increases [22–24], and because GBC is a marker sensitive to target receptor occupancy for drugs [19–21]. To test for this, we took a normative PET map of D1R density from the *neuromaps* repository (using [^11^C]SCH23390 data from Kaller et al. [41]) and again used the *compare_images* command to spatially correlate this map with the map of the paired *t* test results (i.e., the fast versus slow dopamine rate-associated GBC maps). Because we did not have individual-level D1 receptor data for the subjects in this study, we consider this analysis preliminary and include the results in the Supplement.

#### GBC and individual differences in drug ‘high’

Finally, we sought to understand individual differences in the neurocircuitry behind the subjective experience of drug reward. We did not a priori hypothesize a single specific region whose dopamine rate-associated connectivity would be associated with ‘high’, so for exploratory testing we parcellated the GBC maps into large-scale functional networks based on the Cognitive and Affective Neuroscience Lab combined atlas in the *canlab* fMRI toolbox (canlab.github.io). This atlas includes 489 regions that can be aggregated into 23 large-scale networks. Since we did not a priori hypothesize specific subnetworks, we further reduced the number of comparisons by averaging across subnetworks (e.g., Default Mode A, B, and C were averaged to form a single Default Mode network). This resulted in 15 networks for final analysis.

We then performed robust ‘skipped’ correlations [42] between dopamine rate-associated GBC estimates for each large-scale functional network and each individual’s ‘high’ rating in response to MP, using the *Pingouin* package in Python [43]. To get an aggregate single estimate of ‘high’ for each individual, we computed area under the curve of their ‘high’ ratings across the entire 90-min scan, using the composite trapezoidal rule with the *trapz* command in the *numpy* package in Python. Our primary focus for this analysis was on the IV MP (fast dopamine increase) session, since for the oral MP session only 13 participants rated some change in ‘high’ over the course of the scan (rated more than 1 out of 10 for at least one time point). To correct for multiple comparisons across all 15 functional brain networks, we performed Bonferroni correction.

## Results

We previously found that, as expected, dopamine increases to oral MP compared to IV MP started earlier (since oral MP was administered 30 min prior to [^11^C]raclopride whereas IV MP was administered 30 min post [^11^C]raclopride) and were slower and more modest than the fast and strong increases from IV MP. The derivative of the fitted gamma cumulative distribution function to the average delta SUVr(t) across subjects reflects the rate of dopamine increases (Fig. 1C), which we used for subsequent analyses to identify brain circuits that synergized with dopamine dynamics.

**Fig. 2:**
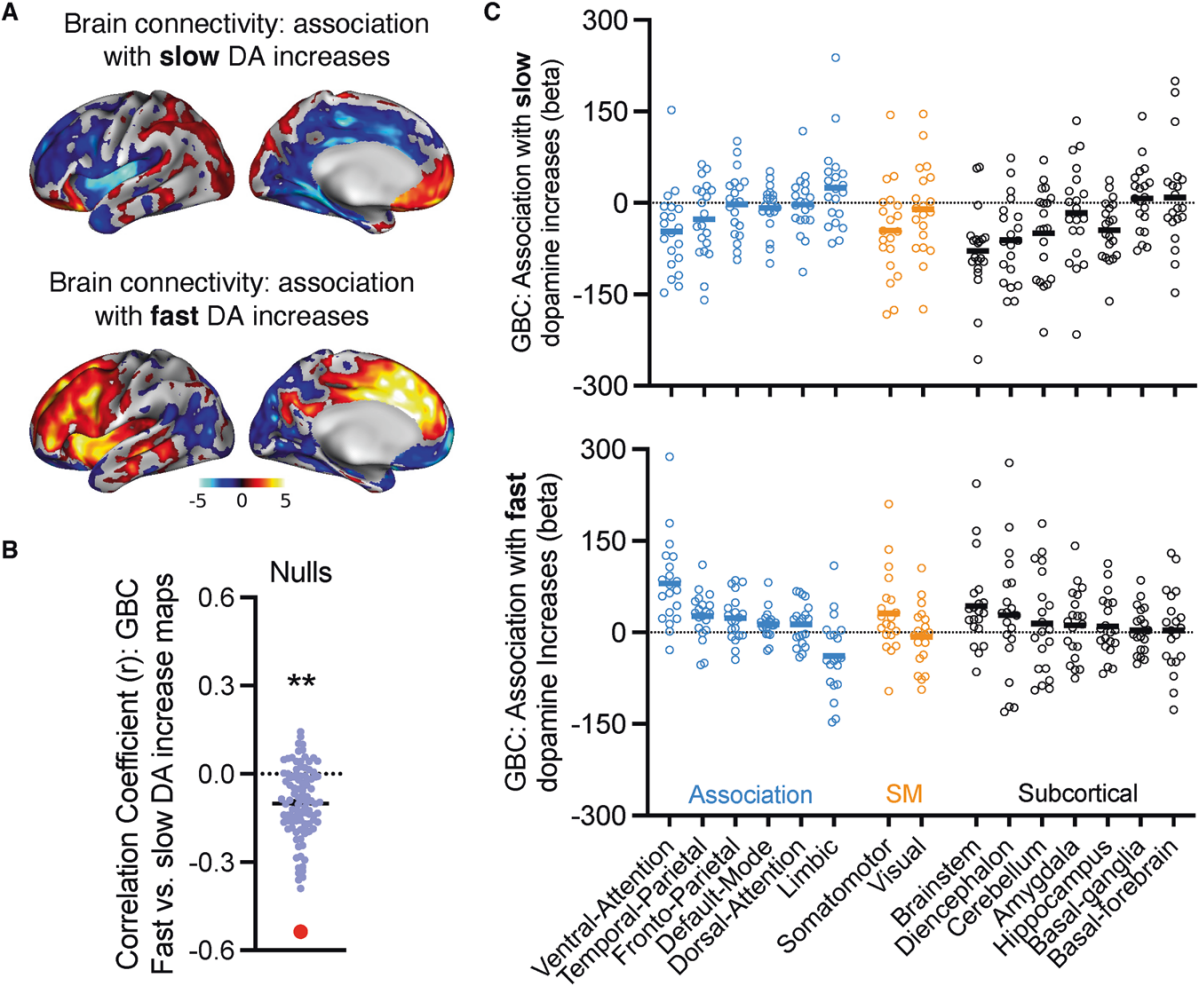
Brain connectivity changes to slow and fast dopamine increases show largely opposing patterns. **A** Group maps (one-sample *t* tests) depicting the fit between global brain connectivity (GBC) and slow (oral methylphenidate; top) or fast (intravenous methylphenidate; bottom) dopamine increase maps. Color bar represents *t* values. **B** The GBC maps to slow versus fast dopamine increases were significantly negatively correlated. Purple dots represent the null distribution (controlling for spatial autocorrelation); red dot is the observed correlation. **C** Parcellation of the maps from panel A into predefined large-scale functional networks. The open circles are beta values for each individual representing the fit between GBC and the speed of dopamine increases. Horizontal lines represent the group mean. Networks were grouped based on whether they generally fall within association cortices (blue); sensorimotor cortices (SM; orange); or subcortical regions (black). For more detailed visualization of these findings throughout the brain, please see Supplementary Fig. S2.

We tested where GBC was significantly associated with the rate of dopamine increases across time. For the regression with slow dopamine increases (oral MP), GBC showed positive associations in ventromedial prefrontal cortex, and negative associations in cingulate, insula, supplementary motor area, dorsomedial and dorsolateral prefrontal cortex, hippocampus, and visual cortex (Fig. 2A, top). For the regression with fast dopamine increases (IV MP), GBC showed largely the reverse pattern: there were negative associations in ventromedial prefrontal cortex and positive associations in cingulate, insula, supplementary motor area, and dorsomedial and dorsolateral prefrontal cortex (Fig. 2A, bottom). Since we hypothesized differential patterns of connectivity to slow versus fast dopamine increases, we formally tested for a spatial anticorrelation between the two GBC maps using spin-based permutation testing; results showed that GBC patterns to slow versus fast dopamine increases were indeed anticorrelated (rho = −0.54, *p*_spin_ < 0.001, controlling for spatial autocorrelation; Fig. 2B). Conjunction analysis revealed that no regions had significant overlap in the connectivity changes to slow and fast dopamine increases. We then examined which functional networks showed the strongest change in association with rate of dopamine increases, for descriptive purposes only. GBC patterns to slow versus fast dopamine increases strongly diverged in several networks, notably the ventral attention network, somatomotor cortex, brainstem and diencephalon (Fig. 2C).

To ensure that these findings were specific to the active drug conditions and not also present in the placebo session, we repeated these analyses using the dopamine rate-associated GBC maps from paired *t* tests (i.e., we performed the spatial correlation of the IV MP > Placebo GBC maps with the Oral MP > Placebo GBC maps). The findings were largely similar to the prior analysis and reported in Supplementary Fig. S3.

We next tested whether fast compared to slow dopamine rate-associated GBC patterns were spatially correlated with dopamine D1 receptor density across the brain. Indeed, the GBC patterns to fast versus slow dopamine increases were significantly positively correlated with a normative map of dopamine D1 receptor availability (rho = 0.22, *p*_spin_ < 0.05, controlling for spatial autocorrelation; Supplementary Fig. S4; Supplementary Table 2).

**Fig. 3:**
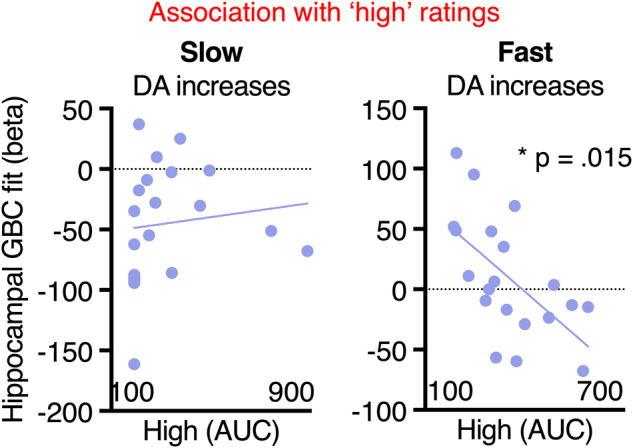
Network GBC changes to speed of dopamine increases and individual differences in subjective ‘high’ to methylphenidate. In exploratory testing, the hippocampus (and no other network) showed a significant correlation between GBC patterns to fast dopamine increases and ‘high’ ratings. The x-axis is the area under the curve (AUC) representing an aggregate measure of the total ‘high’ reported across the scanning session. The y-axis shows beta values for each participant demonstrating the association between fast dopamine increases and GBC. Note: in the oral session only 13 out of 20 participants reported feeling any ‘high’, so there may be floor effects for this session.

Finally, we considered whether GBC patterns to fast dopamine increases were correlated with individual differences in the ‘high’ to intravenous methylphenidate, in exploratory testing. Across all 15 networks, only the hippocampus showed a significant association with ‘high’ ratings (robust ‘skipped’ correlation: *r(*19) = −0.68, Bonferroni-corrected *p* = 0.015; Fig. 3). This association was not present for hippocampal GBC responses to slow dopamine increases (*r(*19) = 0.262, uncorrected *p* = 0.31), though we hesitate to interpret this null finding because only 13 of 20 individuals reported any ‘high’ to oral methylphenidate.

## Discussion

Here we found strikingly divergent patterns of whole-brain functional connectivity to fast versus slow drug-induced dopamine increases. Differences were present in many large-scale brain networks and were spatially aligned with the regional cortical distribution of dopamine D1 receptor availability in the human brain. Further, the degree to which hippocampal connectivity tracked fast dopamine dynamics was associated with the intensity of ‘high’ ratings to MP. We discuss these findings in more detail below.

A growing body of research has characterized the effects of stimulants like methylphenidate on the brain’s functional architecture. Yet findings are disparate, in part because of differences across studies in route of administration, dosage, and analytic strategies. Prior studies examining functional connectivity changes following an acute dose of oral methylphenidate (inducing slow dopamine increases) have reported several findings relevant to our results. A 45 mg dose increased global connectivity in primary motor cortex, and decreased it in lateral prefrontal cortex [44], whereas a 20 mg dose decreased global connectivity density in somatomotor regions including supplementary motor area, and reduced local connectivity density in thalamus [45]. Both 60 mg and 40 mg doses decreased within-network connectivity in sensory and motor cortices [46, 47], though another study that restricted analyses to regions enriched for the dopamine transporter found that a 20 mg dose increased connectivity in motor cortex [48]. In contrast, there are relatively few studies examining functional connectivity following an intravenous dose of a stimulant (inducing fast dopamine increases). A 0.5 mg/kg IV injection of methylphenidate increased connectivity between thalamus and cerebellum in healthy controls and in cannabis users [49]. In contrast, a 0.57 mg/kg dose of IV cocaine (stimulant drug pharmacologically similar to MP) reduced connectivity in primary visual and motor cortex in cocaine users [50]. Similarly, in rhesus monkeys with a history of chronic cocaine administration, self-administration of 0.3 mg/kg IV cocaine broadly decreased global connectivity across the brain [51]. These varied findings suggest that changes in connectivity in response to stimulant drugs are likely to be influenced by prior exposures to those drugs. Additionally, species differences and the effects of self-administration versus experimenter administration might also contribute to divergent findings.

Here, we replicated some of these results and found that connectivity patterns are crucially related to the dynamics of dopamine increases. The oral and IV doses in this study (60 mg and 0.25 mg/kg, respectively) occupy roughly equivalent quantities of striatal dopamine transporters (~70%) in humans [52, 53], and produce roughly the same overall magnitude of dopamine increases [6]. Yet the connectivity patterns to slow and fast dopamine increases were opposing in most networks, and no regions showed significant overlap in connectivity patterns. Some of the global connectivity differences were in a similar direction to those of our prior study on BOLD signal changes, where we found that fast dopamine increases were associated with higher local activity in dorsal anterior cingulate and insula, and lower activity in ventromedial prefrontal cortex [14]. But some different patterns also emerged; for instance, we previously observed that slow dopamine increases were associated with lower BOLD activity in ventromedial prefrontal cortex; whereas here we find that slow dopamine increases were associated with higher global connectivity in this same region. GBC compared to BOLD signal activity also exhibited more widespread differences based on dopamine kinetics: fast compared to slow dopamine increases were associated with greater connectivity in midbrain/brainstem, thalamus, and head of caudate, and lower connectivity in ventral striatum. Notably, some studies found that D1 density is highest in the head of caudate while D2 density is more evenly distributed across the striatum [54, 55], whereas D3 receptors, which have high affinity for dopamine and like D2 receptors are inhibitory, have very high densities in the nucleus accumbens (located in ventral striatum) [56, 57]. The differing spatial distributions of receptor subtypes may explain the opposing connectivity patterns between striatal subregions, as dopamine receptors densities shape functional connectivity within discrete cortico-striatal-midbrain circuits [58–60].

Still, an open question remains: how exactly is GBC sensitive to route of MP administration? Broadly, GBC appears to capture changes in large-scale neuronal communication following drug-induced changes in neurotransmitter binding to receptors [61, 62], a finding that was recently established in studies of 10 different mind-altering drugs including MP [21]. They found that (1) changes in GBC following drug challenges could be predicted based on the distribution of target receptors/transporters and that (2) changes were organized along the unimodal/transmodal gradient hierarchy in neocortex [63]. We likewise found generally divergent patterns along this hierarchy: fast dopamine increases were associated with increased GBC in transmodal association cortices (e.g., dorsolateral prefrontal cortex, insula, dorsal anterior cingulate cortex) and with decreased GBC in primary visual and motor cortices (and vice versa for slow dopamine increases) consistent with the relative cortical distribution of D1 to D2 receptors in the human brain [46]. A notable exception was the ventromedial prefrontal cortex, which showed a GBC pattern opposite to that of other transmodal cortical regions, including an opposite pattern to the association with the regional distribution of D1 receptor availability. Intriguingly the ventromedial prefrontal cortex also has high levels of D3 receptors [64] so its distinct responses might have reflected the relative distribution not just of D1R and D2R but also D3R. We had previously shown that the ventromedial prefrontal cortex was sensitive to expectation effects in healthy participants anticipating to receive IV MP [65], which might have also contributed in the current study to its distinct response from that of other transmodal cortices. Interestingly, in rat infralimbic cortex (a roughly homologous region to the human ventromedial prefrontal cortex), fast infusion of cocaine (5 s injection) in cocaine naïve animals was associated with strong increases in *c-fos* and *arc* mRNA expression, whereas slower infusions (25 s or 100 s injections) did not produce measurable changes [66]. The authors speculated that this may be a critical marker of experience-dependent plasticity that drugs evoke when they enter the brain very quickly and hence have addictive potential. Future preclinical studies could test whether such changes in immediate early gene expression are associated with changes in measures of whole-brain functional network communication such as GBC, as well as the role of the various dopamine receptors in such changes.

Since previous studies found that GBC has strong spatial correspondence with drug-induced changes in target receptor occupancy [19–21], we formally tested the spatial correlation between D1 receptor availability and functional connectivity changes to dopamine rate, in an exploratory analysis (supplement). We reasoned that fast dopamine increases would increase GBC in a pattern spatially aligned with D1 receptor availability, since fast compared to slow dopamine increases would more likely stimulate the low-affinity D1 receptors [22–24]. Indeed, fast but not slow dopamine increases induced global connectivity changes with spatial correspondence to D1 receptors except as discussed before in the ventromedial prefrontal cortex. These data provide preliminary evidence for models on how dopamine receptor occupancy shapes the fMRI response [22] and on our prior work showing that the cortical D1-D2 receptor ratio is strongly associated with functional activity and connectivity responses to MP [46]. They also reinforce support for GBC as a noninvasive measure with strong biological relevance for the effects of acute drug interventions [21, 67]. However, we stress that these findings are preliminary because the D1 receptor maps were derived from normative data outside of the current study. This analysis should therefore be replicated in another sample where D1 receptor data are available for the same individuals who have GBC data.

Out of 15 functional networks tested, only the hippocampus GBC response to fast dopamine increases showed a significant association with intensity of ‘high’ ratings across participants. This was notable because the average GBC association with fast dopamine increases was near zero. However, there was considerable individual variability, and those with decreases in hippocampal GBC tended to experience the strongest ‘high’ to IV MP. The hippocampus may be important for attributing the experience of reward to a particular context; GABAergic inhibitory signals from the hippocampus disinhibit midbrain dopamine neurons and are necessary for cocaine-seeking behavior in rodents [68, 69]. This circuit has also been observed in humans via functional connectivity analysis [70]. Interestingly, when patients with opioid use disorder were inhibiting craving during drug cue exposure, hippocampal activity increased [71]. More broadly, a growing literature suggests that chronic psychostimulant use induces neuroadaptations in the hippocampus that promote relapse vulnerability (for a review, see [72]). For instance, among people with cocaine use disorder in residential treatment, heightened regional cerebral blood flow in the hippocampus (and no other brain region) predicted relapse in the coming month [73]. The current data add to this body of evidence and suggests that hippocampal connectivity during a person’s first intravenous MP exposure may modulate their sensitivity to the rewarding experience from the drug.

Finally, whereas the fast dopamine increases with IV MP were associated with increased connectivity throughout most functional networks it was associated with decreased connectivity in the limbic network; this was a pattern opposite to that observed in association with the slow dopamine increases with oral MP. Since the limbic network comprises the ventromedial prefrontal cortex as well as the temporal pole and the hippocampus, the decreased connectivity of this network is likely to be driven by the distinct response of the ventromedial prefrontal cortex.

There are several limitations to note. First, the cost-intensive nature of this study and its methodological complexity restricted the sample size, which limited our ability to perform certain analyses. For instance, only 13 of 20 individuals reported experiencing any ‘high’ to the oral dose of MP. In future larger samples, it will be interesting to test whether the hippocampus or other networks have a GBC response to slow dopamine increases that explains individual differences in drug reward to oral MP. Second, the dynamic PET data is a relatively noisy measure at the individual subject level compared to the group level, and therefore we were limited to using the average speed of dopamine increases rather than individual-level dopamine measures. Third, our experimental design has relatively low ecological validity since an experimenter-administered drug exposure in a scanner is a different experience than self-administration in a person’s preferred environment. The context of exposure impacts the rewarding properties of drugs [74–76] and the brain dopaminergic response [77–80]. Fourth, oral and IV drug administrations may vary on other unmeasured factors aside from the speed of dopamine increases, such as differences in MP metabolism when administered orally versus IV. However, in a prior analysis of this dataset [6] we confirmed that the oral and IV MP doses elicited similar overall striatal dopamine increases supporting the interpretation that the differences we observed are driven by the rate of dopamine change. Still, IV MP likely elicits a greater peak dopamine increase than oral MP, which we hypothesize could also contribute to greater D1R stimulation and unique connectivity patterns. Peak norepinephrine levels may likewise be higher for IV MP than oral MP. Therefore, while here we estimated connectivity changes based on the rate of dopamine increases to different MP administrations, we cannot definitively rule out that other differences between IV and oral MP played a role in the findings. Lastly, these participants were healthy adults with limited illicit drug use; while the initial subjective response to drugs does seem to be relevant for future chronic use [15] it remains unclear if the brain responses to fast and slow dopamine increases would be similar in individuals with a substance use disorder.

### Supplementary information

Supplement

Consort Flow Diagram

## Data Availability

The deidentified data generated in this study have been deposited in the Open Science Framework (OSF) database and are publicly available at https://osf.io/4qupw/ [81].

## Code availability

Code to produce primary analyses have been deposited in the Open Science Framework (OSF) database and are publicly available at https://osf.io/4qupw/ [81].
