# Supplementary material for: Brain connectivity changes to fast versus slow dopamine increases
Source: Neuropsychopharmacology. 2024 Feb 7;49(6):924–32. doi: 10.1038/s41386-024-01803-8 (PMC11039764; doi:10.1038/s41386-024-01803-8)
Supplement: Supplementary file 1 — Supplement [file 41386_2024_1803_MOESM1_ESM.pdf]

Supplementary material for:

Brain connectivity changes to fast versus slow dopamine increases

Methods

Comparison of ΔSUVr method for estimating 'dynamic dopamine increases' with prior methods.

The SUVr method for estimating dynamic dopamine increases can be seen as an approximation of LSSRM (see demonstration below). LSSRM requires only one scan session with an MP challenge to estimate dynamic dopamine increases, but it necessitates five fit parameters, which hindered reliable quantification of dopamine in our data. This may be in part because we were unable to continuously infuse [^{11}C]raclopride throughout the 90 minutes of scanning (this was due to the challenges posed by the simultaneous PET-MRI setup. Specifically, to ensure safety, our magnetic pump had to be positioned six feet away from the MRI bore. Consequently, utilizing the bolus-plus-infusion method for [^{11}C]raclopride would have necessitated excessively high levels of initial radioactivity (>80mCi), which was deemed unsafe.) Therefore, radioactivity counts were lower at the end of the scan than in a paradigm with a continuous infusion. While LSSRM does not strictly require a paradigm with a continuous infusion, in our dataset we found that the relatively low radioactivity counts made dopamine quantification with LSSRM challenging. However, our design had the advantage of an additional placebo scan for each participant. Therefore, we developed an approach that capitalized on the added reliability the placebo scan affords, and could overcome the lack of a continuous [^{11}C]raclopride infusion. While the ΔSUVr approach requires two scans (MP and placebo) it has an important advantage: it only requires the amplitude of ΔSUVr and the time-to-peak of its derivative for fitting the ΔSUVr data, which improved the reliability of dynamic 'dopamine increases' estimates over prior methods.

The Simplified Reference Tissue Model (SRTM) defines the kinetic $C_T(t)$ of a target region in relation to the kinetic $C_R(t)$ of a reference region[1].

$$C_T(t) = R_1 C_R(t) + k_2 \int_0^t C_R(u)du - k_{2a} \int_0^t C_T(u)du \tag{1}$$

$R_1 = K'_1/K_1$ represents the local rate of delivery in the target tissue compared to the reference tissue, with k_2 representing the transfer rate constant from tissue to blood in the reference region, and k_{2a} representing the transfer rate constant from tissue to blood in the target region. The linear extension of the simplified reference region model (LSSRM[2]) extended this model by incorporating a time-varying efflux rate $k_{2a}(t) = k_{2a} + \gamma\, h(t)$ that accounts for the competition

between the radioligand and the endogenous neurotransmitter at the receptor sites. Here γ represents the magnitude of transient effects and the function $h(t)$ characterizes the endogenous neurotransmitter discharge or an exogenous concurrent drug concentration level. Since MP increases extracellular dopamine, it also increases binding competition and reduces tracer concentration in the target region, Eq [1] can be expressed as:

$$C_T^{MP}(t) = R_1 C_R(t) + k_2 \int_0^t C_R(u)du - k_{2a} \int_0^t C_T(u)du - \gamma \int_0^t C_T(u)h(u)du \tag{2}$$

The standardized uptake value, $SUVr(t)$, is calculated by dividing the uptake value in a specific region of interest (ROI) by the uptake value in a reference region. The reference region is typically an area of the brain that is considered to have minimal specific binding for the radiotracer used in the PET study. The $SUVr$ is used as a simplified way to quantify the relative accumulation or binding of a radiotracer in a particular brain region compared to the reference region.

$$SUVr(t) = \frac{C_T(t)}{C_R(t)} \tag{3}$$

The $SUVr$ is beneficial because it allows for comparison and analysis of PET data across different individuals or studies by normalizing the values to a reference region. This normalization accounts for potential variations in overall radiotracer uptake due to factors such as individual differences in blood flow or metabolism.

The $SUVr$ change, $\Delta SUVr(t)$, caused by MP-related increases in endogenous dopamine quantifies the change in radiotracer binding with respect to the placebo condition.

$$\Delta SUVr(t) = \frac{C_T(t) - C_T^{MP}(t)}{C_R(t)} \tag{4}$$

Inserting [1] and [2] in [4] $\Delta SUVr(t)$ can be expressed as

$$\Delta SUVr(t) = \frac{\gamma \int_0^t C_T(u)h(u)du}{C_R(t)} \tag{5}$$

The instantaneous tissue concentration in the reference region $C_R(t)$ is described by the operational equation of the one-tissue compartment model:

$$\frac{dC_R(t)}{dt} = K_1 C_p(t) - k_2 C_R(t), \tag{6}$$

where the uptake rate constant $K_1 = 0.092$ mL/min.g and $k_2 = 0.45$ min^{-1} (see ref.[2]) The plasmatic input function can be represented by the tri-exponential function

(7)

$$C_p(t) = \begin{cases} \frac{(A_1+A_2+A_3)}{t_{peak}} t & if\ t < t_{peak} \\ \sum_{i=1}^{3} A_i \exp\left(-\frac{\ln(2)}{T_i}\left(t - t_{peak}\right)\right) & if\ t \geq t_{peak} \end{cases},$$

with $\vec{A} = (A_1, A_2, A_3) = (288.6,1.1,409.7) Bq/ml$, $\vec{T} = (T_1, T_2, T_3) = (4.28,735.5,183.5) sec$, and t_{peak}= 110 sec (see ref.[3]). The concentration of the tracer in the striatum can be simulated using specific parameters (R_1=1.154, and k_{2a}=0.065 min^{-1}) and Eq [1]. **Supplementary Figure 1A** shows that $C_R(t)$ peaks earlier than $C_T(t)$, which reaches a maximum near the MP injection time (t = 30 min). Additionally, Eq [5] can be approximated as:

$$\Delta SUVr(t) \propto \int_0^t h(u) du \tag{8}$$

where $h(t)$ was modelled by a gamma probability distribution function (**Supplementary Figure 1B**)[4]. A high correlation (r = 0.987) between $\Delta SUVr(t)$ and $\int_0^t h(u) du$ was obtained in a 60 min window centered at the time of MP injection (**Supplementary Figure 1C,D**). This first order approximation shows that $\Delta SUVr(t)$ is proportional to the accumulation of endogenous dopamine caused by MP, which in our approach is represented by $F(t)$, and that the instantaneous dopamine change $h(u)$ is equivalent to the rate of dopamine, which in our approach is represented by $f(t)$.

The ΔSUVr approach offers a significant advantage over prior methods, such as LSSRM, by eliminating the need for individual-specific SRTM parameters (R_1, k_2, k_{2a}) to estimate dopamine increases. This enhances the robustness of model fitting as it only requires the amplitude of ΔSUVr and the time-to-peak of its derivative for fitting the ΔSUVr data.

References

1. Lammertsma, A. A. & Hume, S. P. Simplified Reference Tissue Model for PET Receptor Studies. *NeuroImage* **4**, 153–158 (1996).

2. Alpert, N. M., Badgaiyan, R. D., Livni, E. & Fischman, A. J. A novel method for noninvasive detection of neuromodulatory changes in specific neurotransmitter systems. *NeuroImage* **19**, 1049–1060 (2003).

3. Irace, Z. *et al.* Bayesian Estimation of the ntPET Model in Single-Scan Competition PET Studies. *Frontiers in Physiology* **11**, (2020).

4. Normandin, M. D., Schiffer, W. K. & Morris, E. D. A linear model for estimation of neurotransmitter response profiles from dynamic PET data. *NeuroImage* **59**, 2689–2699 (2012).

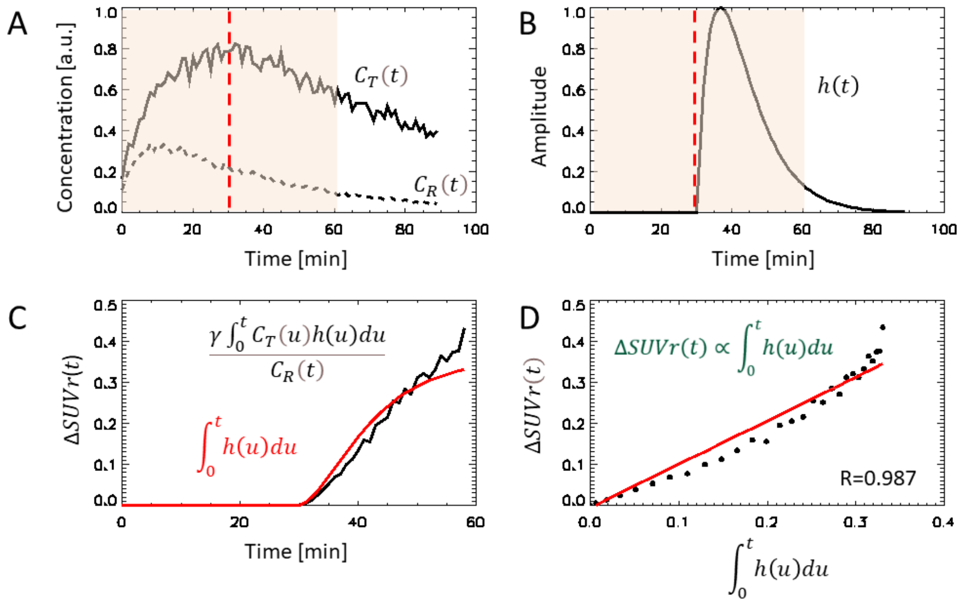

Figure S1.

Simulations demonstrating the similarity between the $\Delta SUVr$ method used in the current study to estimate dynamic dopamine increases, and the 'linear simplified reference region model' (LSSRM) method that has been used in prior studies. A) Time-varying concentrations of [11C]raclopride in the striatum, $C_T(t)$, and in the cerebellum, $C_R(t)$. B) A gamma variate function modeling the endogenous dopamine increases elicited by methylphenidate (MP), $h(t)$. C) Dynamics of SUVr changes, $\Delta SUVr(t)$, caused by MP-related increases in endogenous dopamine modeled with the LSSRM (black) and the approximation used in this study. D) Linear association between the exact and approximated LSSRM solutions. Normal random noise (5%) was added to $C_R(t)$ and $C_T(t)$. Dashed lines indicate the time of MP injection.

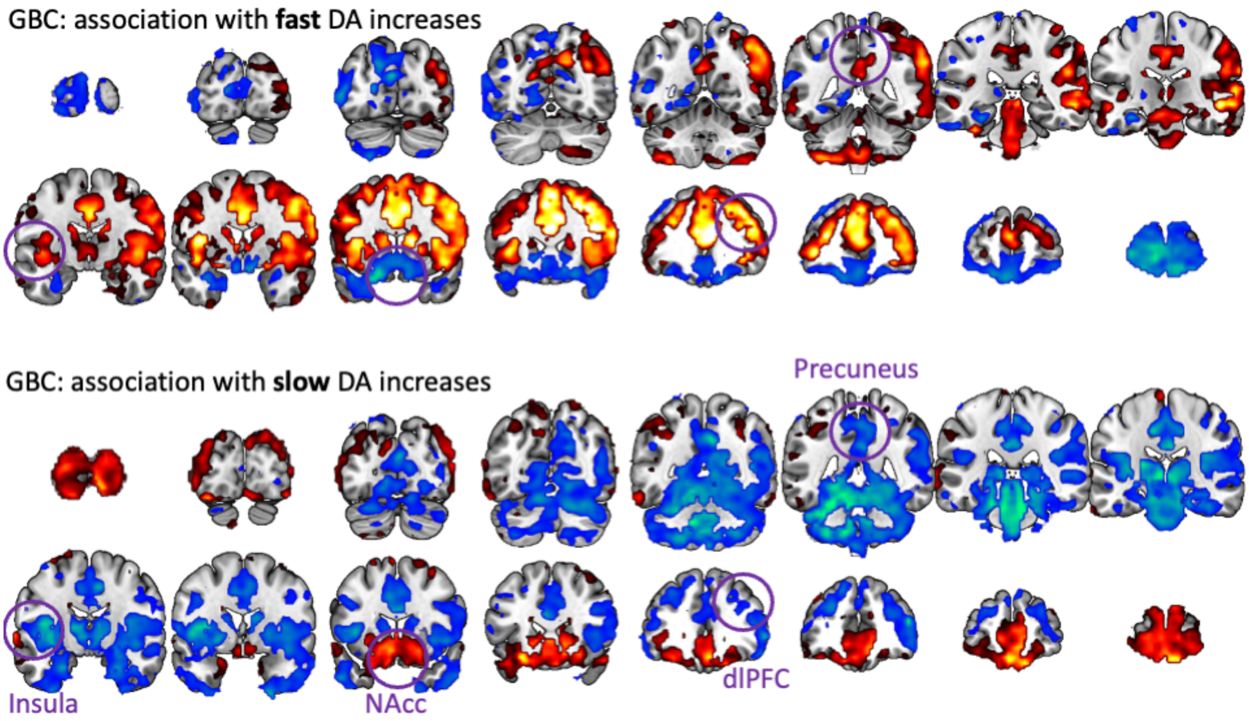

Figure S2.
Brain connectivity changes to slow and fast dopamine increases show largely opposing patterns. Group maps (one-sample t-tests) depicting the fit between global brain connectivity (GBC) and fast (intravenous methylphenidate; top) or slow (oral methylphenidate; bottom) maps. These results are the same as presented in Figure 2A of the main manuscript; here we are showing more detailed maps overlaid on coronal brain slices. We also highlight several notable regions, in purple circles, where connectivity patterns diverged: Precuneus, Insula, Nucleus Accumbens (NAcc) and dorsolateral prefrontal cortex (dlPFC).

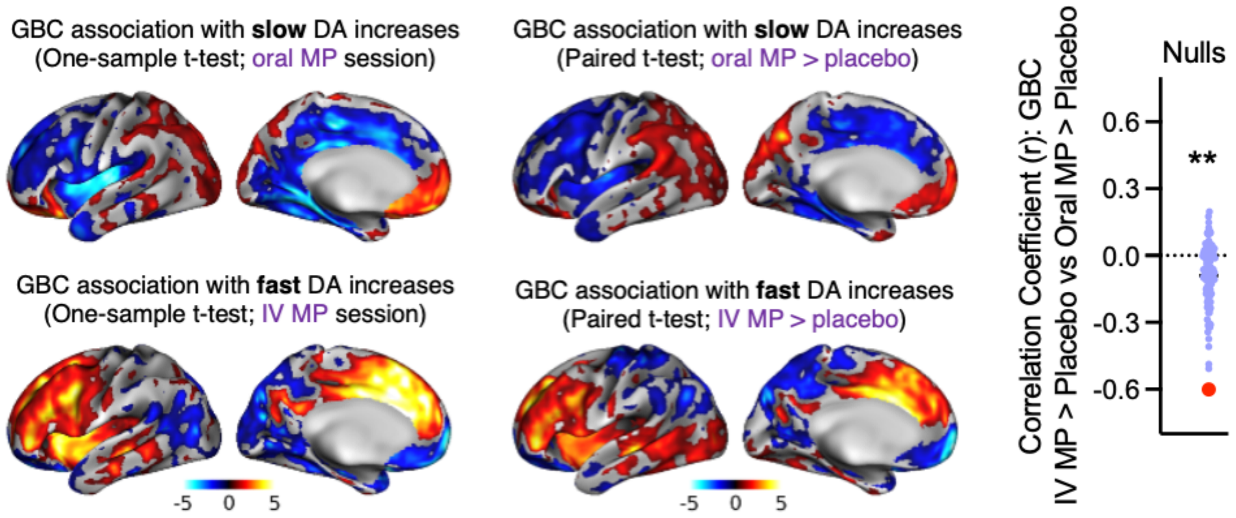

Figure S3.

Placebo control analysis: brain connectivity changes to slow and fast dopamine increases show largely opposing patterns. Left column: Group maps (one-sample t-tests) depicting the fit between global brain connectivity (GBC) and slow (oral methylphenidate (MP); top) or fast (IV MP; bottom) dopamine (DA) increase maps (identical to **Figure 2A** of main manuscript). Middle column: additional control analyses directly comparing the speed of DA increases in the active drug conditions to that of the placebo condition (i.e., paired t-tests representing IV MP > Placebo and Oral MP > Placebo). These results were highly similar to the original analyses (Supplementary Figure S2) demonstrating specificity of the findings to the active drug conditions. Right column: The GBC maps to oral MP > placebo versus IV MP > placebo were significantly negatively correlated. Purple dots represent the null distribution (controlling for spatial autocorrelation); red dot is the observed correlation (rho = -.60; $p_{spin} < .001$). Thus, the primary finding of opposing connectivity patterns to slow and fast DA increases in the main manuscript (**Figure 2B**) was preserved when controlling for placebo effects. Color bars represent *t*-values.

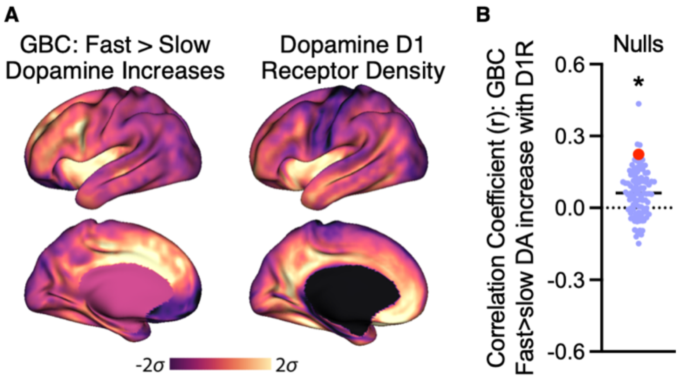

Figure S4.

Dynamic global brain connectivity (GBC) changes with dopamine rate and association with the cortical distribution of dopamine D1 receptors. A) Left: direct comparison (paired *t*-test) of GBC associations with fast versus slow dopamine increases to methylphenidate. Right: normative PET map of D1 receptor density (using [^{11}C]SCH23390 data from the *neuromaps* repository). B) Spatial permutation test demonstrating a significant positive spatial correlation between "fast > slow" GBC associations and D1 receptor density. Purple dots represent the null distribution (controlling for spatial autocorrelation); red dot is the observed correlation.

Supplementary Table 1. Demographic summary for the participants (n = 20 healthy adults).

**Age**	Mean ± SD	36.07 ± 9.80
**Sex**	n, Male/Female (%)	11/9 (55/45)
**BMI**	Mean ± SD	26.96 ± 2.66
**IQ**	Mean ± SD	108.7 ± 11.6
**Race**	n, White (%)	6 (30)
	n, Black/AA (%)	10 (50)
	n, Asian (%)	2 (10)
	n, Other (%)	2 (10)

Supplementary Table 2. Significant clusters for the analysis: global brain connectivity changes to fast versus slow dopamine increases (paired *t*-test; see **Figure S3A, left**). Note: dACC = dorsal anterior cingulate cortex; PFC = prefrontal cortex; OFC = Orbitofrontal cortex; PLA = placebo; FWE = familywise error correction; FDR = false discovery rate correction; MNI = Montreal Neurological Institute

Region	pFWE	pFDR	k	peak T	peak Z	Coordinates (MNI)
**Fast > Slow Dopamine Increases (IV MP > Oral MP)**						
dACC	< .001	< .001	1221	10.06	5.85	[-3 20 20]
				7.73	5.14	[-6 29 17]
				7.22	4.95	[-6 14 32]
Insula Left	< .001	< .001	436	9.46	5.69	[-36 5 5]
				7.49	5.05	[-33 -4 11]
				7.47	5.04	[-39 -10 11]
Brainstem	< .001	< .001	937	8.80	5.49	[6 -34 -7]
				7.99	5.23	[-3 -25 2]
				7.81	5.17	[6 -34 -16]
PFC Right	< .001	< .001	1000	6.73	4.76	[45 44 11]
				6.52	4.67	[54 32 -1]
				6.49	4.66	[39 17 23]
PFC Left	0.020	0.014	68	6.36	4.60	[-39 5 29]
				5.26	4.08	[-45 2 23]
				4.64	3.75	[-42 14 29]
Anterior PFC Left	0.009	0.007	82	5.83	4.36	[-27 26 29]
				5.28	4.09	[-21 41 23]
				5.00	3.95	[-27 44 11]
Caudate/Thalamus	0.004	0.004	96	5.70	4.30	[15 5 11]
				5.50	4.20	[9 -1 17]
				4.05	3.39	[15 -16 23]
Precuneus	0.023	0.014	66	5.61	4.26	[6 -52 35]
				4.07	3.41	[3 -46 29]
**Slow > Fast Dopamine Increases (Oral MP > IV MP)**						
Nucleus Accumbens	< .001	< .001	319	7.49	5.05	[-12 20 -7]
				6.68	4.74	[9 14 -22]
				6.00	4.44	[12 20 -13]
OFC/Frontopolar	< .001	< .001	467	7.06	4.89	[9 65 -22]
				6.69	4.74	[0 56 -22]
				5.96	4.42	[-6 68 5]
