# Supplementary material for: Brain connectivity changes to fast versus slow dopamine increases
Source: Neuropsychopharmacology. 2024 Feb 7;49(6):924–32. doi: 10.1038/s41386-024-01803-8 (PMC11039764; doi:10.1038/s41386-024-01803-8)
Supplement: Supplementary file 2 — Consort Flow Diagram [file 41386_2024_1803_MOESM2_ESM.doc]

Assessed for eligibility

(n = 25)

**Enrollment**

Excluded (n = 0)

Randomized (n = 25)

#

**Allocation**

**Follow up**

**Analysis**

Allocated to intervention

(n = 25)

Received allocated intervention (n = 25)

Did not receive allocated intervention (n = 0)

Discontinued intervention (n = 5) -- Technical problems with scan acquisition

Analyzed (n = 20)

Excluded from analysis

(n = 0)
